# Metabolic engineering of *Corynebacterium glutamicum* for producing branched chain amino acids

**DOI:** 10.1186/s12934-021-01721-0

**Published:** 2021-12-24

**Authors:** Shengzhu Yu, Bo Zheng, Zhenya Chen, Yi-Xin Huo

**Affiliations:** Key Laboratory of Molecular Medicine and Biotherapy, School of Life Science, Beijing Institute of Technology, No. 5 South Zhongguancun Street, Haidian District, Beijing, 100081 China

**Keywords:** *Corynebacterium glutamicum*, BCAAs biosynthesis, Metabolic engineering, Feedback inhibition, Acetohydroxy acid synthase

## Abstract

**Background:**

Branched chain amino acids (BCAAs) are widely applied in the food, pharmaceutical, and animal feed industries. Traditional chemical synthetic and enzymatic BCAAs production in vitro has been hampered by expensive raw materials, harsh reaction conditions, and environmental pollution. Microbial metabolic engineering has attracted considerable attention as an alternative method for BCAAs biosynthesis because it is environmentally friendly and delivers high yield.

**Main text:**

*Corynebacterium glutamicum* (*C. glutamicum*) possesses clear genetic background and mature gene manipulation toolbox, and has been utilized as industrial host for producing BCAAs. Acetohydroxy acid synthase (AHAS) is a crucial enzyme in the BCAAs biosynthetic pathway of *C. glutamicum*, but feedback inhibition is a disadvantage. We therefore reviewed AHAS modifications that relieve feedback inhibition and then investigated the importance of AHAS modifications in regulating production ratios of three BCAAs. We have comprehensively summarized and discussed metabolic engineering strategies to promote BCAAs synthesis in *C. glutamicum* and offer solutions to the barriers associated with BCAAs biosynthesis. We also considered the future applications of strains that could produce abundant amounts of BCAAs.

**Conclusions:**

Branched chain amino acids have been synthesized by engineering the metabolism of *C. glutamicum*. Future investigations should focus on the feedback inhibition and/or transcription attenuation mechanisms of crucial enzymes. Enzymes with substrate specificity should be developed and applied to the production of individual BCAAs. The strategies used to construct strains producing BCAAs provide guidance for the biosynthesis of other high value-added compounds.

## Introduction

Branched chain amino acids (BCAAs), including l-valine, l-leucine, and l-isoleucine, are essential amino acids [1, 2] that are widely used in the food [3], pharmaceutical [4, 5], and animal feed [6] industries. The global market for BCAAs during 2020 was USD 233 million, and this is likely to reach USD 303 million by the end of 2026, due to a compound annual growth rate of 3.80% [7]. The market proportions of valine, leucine, and isoleucine are, respectively, 48%, 34%, and 18% [8]. Generally, the consumption of a leucine to isoleucine to valine ratio of 2:1:1 before, during, or after training to initiate muscle protein synthesis, increase energy, and reduce fatigue has dominated the market (87.1%) [9]. Increasing demand for large amounts of BCAAs has driven the need for more rapid and efficient methods of production. Traditional BCAAs production methods mainly include chemical synthesis, protein hydrolysis, and enzymatic catalysis in vitro [10, 11]. However, the raw materials for these methods are expensive, reaction conditions are harsh, and such reactions pollute the environment. Microbial-based metabolic engineering has recently emerged as an economical and practical tool for the production of high-value compounds [12]. Compared with conventional methods, microbial-based metabolic engineering offers many advantages such as inexpensive raw materials, eco-friendly fermentation conditions, and high yield.

Gram-positive *Corynebacterium glutamicum* (*C. glutamicum*) is regarded as a model organism, thanks to an established genetic background and a corresponding mature gene manipulation toolbox [13]. It is also generally regarded as safe (GRAS) [14]. Thus, *C. glutamicum* has served as a host for producing industrial-scale medicines, as well as bulk and food-grade chemicals. Food-grade glutamic acid, lysine, and BCAAs have been biosynthesized by *C. glutamicum* using various strategies [6, 15, 16]. Traditional *C. glutamicum* strains have produced industrial amounts of BCAAs via random mutagenesis and screening. However, random mutations in the *C. glutamicum* genome can result in physiological changes, growth stagnation, and the accumulation of toxic by-products [6]. The desirable properties induced by random mutations could not be sustained over multiple generations, which resulted in a decreased capacity for producing BCAAs. Therefore, strains were needed that could stably generate industrial quantities of BCAAs.

Generally, rationally designed host strains achieve stable performance and generate good-quality products. Based on its genetic background and its mature gene manipulation toolbox, *C. glutamicum* has been rationally engineered to boost BCAAs production. Acetohydroxy acid synthase (AHAS) is a crucial enzyme in the *C. glutamicum* BCAAs biosynthetic pathway, but it is susceptible to feedback inhibition by BCAAs [17]. The many modifications of this enzyme and their corresponding effects are summarized and reviewed herein. We analyzed the importance of AHAS modification to production ratios of l-valine, l-leucine, and l-isoleucine. We then considered a possible catalytic mechanism of AHAS and investigated the future directions of AHAS modifications. We also summarized other engineering strategies to enhance BCAAs production such as the overexpression or protein engineering of rate-limiting enzymes, knocking down or knocking out competing pathways, or regulating the balance of entire pathways. The advantages and disadvantages of each strategy are comprehensively discussed. Based on the outcomes of extant strategies, we suggest others that might further increase BCAAs production. We investigated future applications of high-performance strains in the biosynthesis of BCAAs derivatives, development of BCAAs biosensors, and a possible applicable regulation strategy.

## Metabolic pathways of BCAAs in *C. glutamicum*

**Fig. 1:**
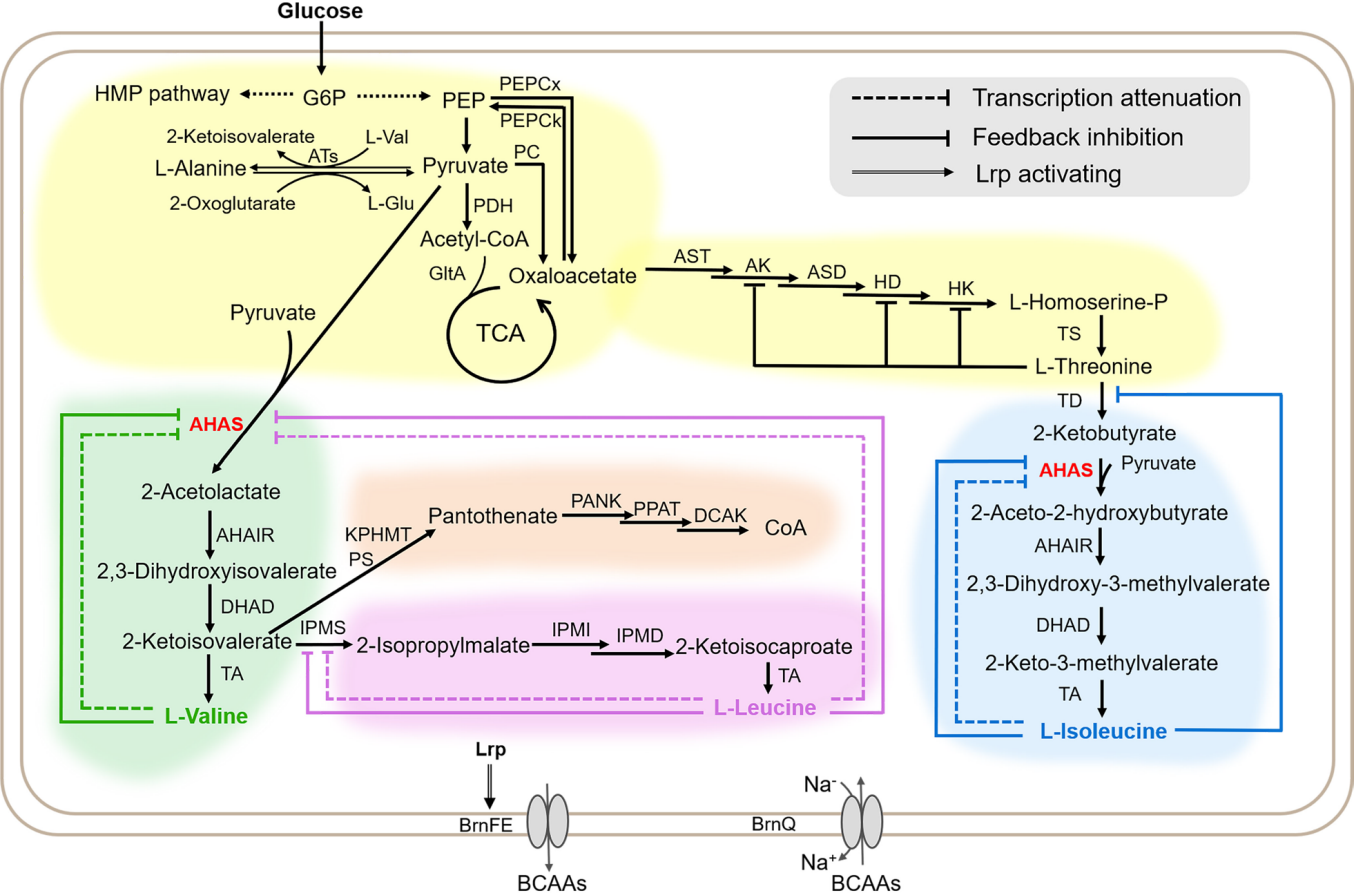
Biosynthetic pathway and regulations of BCAAs in *C*. *glutamicum*. The green areas represent l-valine metabolic pathway, the pink areas represent l-leucine metabolic pathway, the blue areas represent l-isoleucine metabolic pathway, the yellow areas represent central metabolic pathway and l-threonine metabolic pathway, the orange areas represent pantothenate metabolic pathway. The terminating symbol of dotted lines indicate transcription attenuation, the terminating symbol of solid lines indicate feedback inhibition, and the double-line arrow indicates Lrp activation. PEPCx: PEP carboxylase; PEPCk: PEP carboxykinase; PC: pyruvate carboxylase; ATs: aminotransferase; PDH: pyruvate dehydrogenase; GltA: citrate synthase; AST: Aspartate aminotransferase; AK: aspartate kinase; ASD: semialdehyde dehydrogenase; HD: homoserine dehydrogenase; HK: homoserine kinase; TS: threonine synthase; TD: threonine dehydratase; AHAS: acetohydroxy acid synthase; AHAIR: acetohydroxy acid isomerase; DHAD: hydroxy acid dehydratase; TA: branched-chain amino acid aminotransferase; IPMS: isopropyl malate synthase; IPMI: α-isopropylmalate isomerase; IPMD: α-isopropylmalate dehydrogenase; Lrp: leucine-responsive protein; KPHMT: ketopantoate hydroxymethyltransferase; PS: pantothenate synthetase; PANK: pantothenate kinase; PPAT: phosphopantetheine adenylyltransferase; DCAK: dephospho-CoA kinase

Figure 1 shows the biosynthetic pathways of BCAAs in *C. glutamicum*. l-valine, l-leucine, and l-isoleucine are all derived from the central metabolite, pyruvate. Their production pathways share crucial enzymes such as acetohydroxy acid synthase (AHAS), acetohydroxy acid isomeroreductase (AHAIR), dihydroxy acid dehydratase (DHAD), and aminotransferase (TA). Two pyruvate molecules are catalyzed by AHAS to produce 2-acetolactate, which is sequentially catalyzed by AHAIR and DHAD to produce 2-ketoisovalic acid (2-KIV). Aminotransferase subsequently catalyzes 2-KIV to l-valine. Isopropyl malate synthase (IPMS) competes with TA for 2-KIV and catalyzes 2-KIV to 2-isopropylmalate, which is then sequentially converted to 2-ketoisocaproate by α-isopropylmalate isomerase (IPMI) and α-isopropylmalate dehydrogenase (IPMD). Thereafter, 2-ketoisocaproate is catalyzed by TA to generate l-leucine. Oxaloacetate derived from phosphoenolpyruvate or pyruvate undergoes multi-step enzymatic catalysis to produce l-threonine, which is catalyzed by threonine dehydratase (TD) to 2-ketobutyrate, which along with pyruvate, is catalyzed by AHAS to generate 2-aceto-2-hydroxybutyrate that is sequentially catalyzed by AHAIR, DHAD, and TA to biosynthesize l-isoleucine.

Feedback derived from l-valine, l-leucine, or l-isoleucine accumulation inhibits AHAS activity [18], the expression of which is regulated by transcriptional attenuation of these amino acids. Furthermore, TD activity is inhibited by feedback derived from l-isoleucine accumulation [19]. The activity and expression of IPMS are, respectively, regulated by leucine feedback inhibition and leucine transcription attenuation [20]. l-threonine accumulation inhibits the activity of enzymes homoserine kinase (HK), homoserine dehydrogenase (HD), and aspartate kinase (AK), which participate in l-threonine biosynthetic pathway [21]. The transport proteins BrnFE and BrnQ are, respectively, responsible for transporting BCAAs from cells into the extracellular milieu and *vice-versa*. The expression of BrnFE is positively regulated by the global transcription factor Lrp.

## Modification of AHAS to enhance BCAAs production

### Properties and catalysis of AHAS

Acetohydroxy acid synthase plays significant roles in the BCAAs biosynthetic pathway by inducing the crucial catalysis of two pyruvate molecules to produce 2-acetolactate, and that of 2-ketobutyrate and pyruvate to generate 2-aceto-2-hydroxybutyrate. The *k*_cat_/*K*_m_ of AHAS is significantly higher towards 2-ketobutyrate and pyruvate than towards the original two pyruvate molecules [22]. Catalysis by AHAS requires thiamine pyrophosphate (ThDP), divalent metal ions, and flavin adenine dinucleotide (FAD), for which it has high affinity [23–25]. Thiamine pyrophosphate directly participates in the catalytic reaction of AHAS and combines with it to form the reaction intermediate, hydroxyethyl-ThDP.

Acetohydroxy acid synthase is a tetramer comprising two large (~ 60 kDa) catalytic and two small (~ 10–17 kDa) regulatory subunits [26, 27] that are encoded by *ilvB* and *ilvN*, respectively [28]. The *ilvBNC* operon contains the *ilvB* and *ilvN* genes [29] and the 15-amino-acid leading peptide *ilvL* (MTIIRLVVVTARRLP*) between the promoter and the expressed genes. The leading peptide can attenuate the transcription of *ilvB* and *ilvN* in the presence of BCAAs [30]. The catalytic subunit of AHAS is usually regulated by BCAAs binding to the regulatory subunit, which then inhibits the activity of AHAS [31]. The IC_50_ values of AHAS for l-valine, l-leucine, and l-isoleucine are 0.110, 0.790 and 0.410 g/L, respectively, indicating that l-valine is the most powerful inhibitor of AHAS activity among these amino acids [32].

### Removing feedback inhibition and transcriptional attenuation of AHAS

AHAS have been engineered to relieve or weaken the feedback inhibition and transcription attenuation of AHAS, with positive results. For example, the amino acids G-I-I at positions 20, 21, and 22 of the regulatory subunit *ilvN* have been mutated into D-D-F to generate the M13 mutant that resisted feedback inhibition by 1.17 g/L l-valine, l-isoleucine, or l-leucine [27]. Thereafter, mutant M13 was introduced into *C. glutamicum*, which increased the leucine titer from 3.46 g/L to 7.21 g/L [33]. Site-directed mutagenesis at the conserved regions of *ilvN*, such as A42V, A89V, or K136E also improves AHAS resistance to BCAAs. The mutant A42V/A89V maintained > 93% activity in the presence of 1.27 g/L BCAAs. Incorporating this mutant into the BCAAs biosynthetic pathway increased l-valine production from 1.95 to 10.1 g/L [34]. In addition, truncating 53 amino acids from the C-terminus of *ilvN* generated a shorter AHAS that could maintain 90.8% activity in the presence of 0.590 g/L exogenous l-valine [35].

Besides introducing mutations into the regulatory subunit, the catalytic subunit *ilvB* has been mutated in an attempt to decrease the feedback inhibition of AHAS by BCAAs. For instance, *ilvB* harboring the W503Q mutation increased l-leucine production to 3.40 g/L, which was 1.1-fold more than that in wild-type *ilvB*. W503Q and T96S mutations in *ilvB* resulted in a 1.2-fold increase in the l-valine titer (3.40 g/L) compared with that in wild-type *ilvB* [36]. Table 1 summarizes the AHAS modifications and their results.

**Table 1:** Modification of AHAS for BCAAs production

Host	Mutation sites of AHAS	Titer of BCAAs (shake flask) (g/L)	Substrate	References
*C. glutamicum* ATCC13032	G20D/I21D/I22F	l-Valine: 15.2	Glucose	[27]
*C. glutamicum* A-1	Truncating 53 amino acids from *ilvN* C-terminus	l-Valine: 11.2	Glucose	[35]
*C. glutamicum* ATCC14067	A42V/A89V	l-Valine: 10.1	Glucose	[34]
*C. glutamicum* KCCM11201P	W503Q/T96S	l-Valine: 3.40	Glucose	[36]
*C. glutamicum* KCCM11662P	W503Q	l-Leucine: 3.40	Glucose	[36]

Studies have so far partly addressed the feedback inhibition of BCAAs towards AHAS, and while BCAAs production has been somewhat improved, feedback inhibition was not completely eliminated. Therefore, AHAS still requires engineering using other strategies. For example, considering the non-conservative property of regulatory subunits, the regulatory subunit of *C. glutamicum* AHAS might be substituted with the regulatory subunit of *Escherichia coli* AHAS II, which has no feedback inhibition by l-valine and does not influence AHAS activity [6]. This engineered AHAS might be useful for l-valine production.

In addition to releasing feedback inhibition, increasing AHAS activity was also deemed important. The catalytic subunit of AHAS would be an appropriate modification target to improve AHAS activity because it catalyzes reactions. Future investigation might search for and compare significant regions or sites of AHAS in *C. glutamicum* with those of other species and then mutagenize them to boost AHAS activity. Combining modified regulatory and catalytic subunits might result in an AHAS mutant with powerful catalytic activity and resistance to feedback inhibition.

### Analyzing the significance of AHAS substrate specificity

*Corynebacterium glutamicum* AHAS catalyzes the condensation of two pyruvate molecules (3–3 addition reaction) in the l-valine and l-leucine biosynthetic pathways and of one molecule each of pyruvate and 2-ketobutyrate (3–4 addition reaction) in the l-isoleucine pathway [37]. Native AHAS from *C. glutamicum* displays higher affinity to 2-ketobutyrate than to pyruvate. Therefore, *C. glutamicum* is inclined to accumulate l-isoleucine, which is synthesized with a precursor of 2-ketobutyrate [22, 27]. In addition, native AHAS isozyme II from *E. coli* represents a 60-fold higher specificity for 2-ketobutyrate than for pyruvate [38]. The substrate promiscuity of AHAS results in the simultaneous production of three BCAAs, but other by-products are generated that redirect carbon flux and waste carbon sources. The end result is a decrease in target BCAAs production. The substrate selectivity of AHAS towards pyruvate or 2-ketobutyrate determines the production ratios of l-valine, l-leucine, and l-isoleucine. Fermentation products of the three BCAAs with suitable ratios might meet the needs of practical direct application to the food or feed industries. For example, the market has the greatest demand for a ratio of 2:1:1 ratio of l-leucine, l-isoleucine, and l-valine. However, the structure and performance similarity of these BCAAs hinder their separation and purification. Hence, modifying AHAS to produce individual BCAAs has significantly decreased the costs of isolation and purification.

## Regulation of metabolic flux of BCAAs production pathways

### Regulation of central carbon metabolism

Various strategies have been applied to direct a carbon source towards a target biosynthetic pathway to efficiently improve its metabolic flux and further enhance target compound production. More carbon sources can be introduced into BCAAs production pathways. Central metabolic pathways, including glycolysis, the TCA cycle, and the pentose phosphate pathway act as key nodes to balance cell growth and target compound production. Pyruvate generated by the glycolytic pathway is an important intermediate for powering cell growth and an essential precursor for BCAAs biosynthesis in *C. glutamicum* [39]. Hence, regulating pyruvate accumulation can redirect carbon sources for BCAAs biosynthesis. The amount of pyruvate accumulated depends on regulation of the expression of pyruvate metabolism-related enzymes. For example, knocking out the *aceE* gene encoding pyruvate dehydrogenase (PDH), which converts pyruvate into the precursor for TCA cycle (acetyl-CoA), enhances pyruvate accumulation and increases the carbon flux to produce l-valine. With *ilvBNCE* overexpression, *C. glutamicumΔaceE* can produce 22.8 g/L l-valine through fed-batch fermentation in acetate medium [40]. Subsequently, the *pqo* gene encoding pyruvate/quinine oxidoreductase (Pqo) that catalyzes the conversion of pyruvate to acetate has been knocked out in *C. glutamicumΔaceE*. This strain can accumulate 26.4 g/L l-valine within 24 h [41]. In addition, replacing the native *aceE* promoter with the weak A16 promoter decreases the transcriptional level of *aceE*. This increased the fermentation yield of l-valine from 0.071 to 0.241 g/g glucose, with a corresponding valine titer of 10.5 g/L. The *pqo* and *ppc* genes were subsequently deleted in *C. glutamicum/aceE A16* to reduce pyruvate flowing into the synthetic pathways of other compounds; this strain produced ~ 86.5 g/L l-valine after fed-batch fermentation [42]. Moreover, knocking out *pyc* in *C. glutamicum* to reduce pyruvate flowing into the TCA cycle increased the production of l-valine and l-leucine from 5. 93 to 6.74 and 15.7 to 16.9 g/L, respectively [43]. Citrate synthase (GltA) in *C. glutamicum* catalyzes the entry of acetyl-CoA into the TCA cycle. Substituting the native *gltA* promoter with a weaker promoter P_*dapA*-L1_, decreases GltA activity by 16% and increases l-leucine production to 6.82 g/L [33]. Replacing the native *aceE* and *gltA* promoters with the growth-regulated P_CP_2836_ promoter improved l-valine production at the stationary phase [44]. Table 2 summarizes these examples.

**Table 2:** Regulation of central carbon metabolism in BCAAs producing strains

Host	Overexpression genes	Knockout genes	Titer of BCAAs (shake flask) (g/L)	Titer of BCAAs (fed batch) (g/L)	Productivity (g/(L × h))	Yield (g/g glucose)	Substrate	References
*C. glutamicum aceE* A16	*ilvBNC*, *ilvE*	Δ*pqo*, Δ*ppc*		l-Valine: 86.5	1.60	0.234	Glucose	[42]
*C. glutamicum* ATCC13869	*ilvBNC*, *lrp*, *brnFE*	Δ*aceE*, Δ*alaT*, Δ*ilvA*	l-Valine: 28.5	l-Valine: 51.2	0.533	0.308	Glucose	[45]
*C. glutamicum* ATCC13032	*ilvBNC*, *ilvE*	Δ*aceE*, Δ*pqo*, Δ*pgi*		l-Valine: 48.0	0.656	0.488	Glucose, acetate	[41]
*C. glutamicum* ATCC13032	*ilvBNC*, *ilvE*	Δ*aceE*	l-Valine: 12.3	l-Valine 24.6	1.17	0.391	Glucose, acetate	[40]
*C. glutamicum* AN02		ΔP_*gltA*_:: P_CP_2836_	l-Valine: 15.3			0.168	Glucose	[44]
*C. glutamicum* ATCC13032	*ilvBNC*, *ilvE*	Δ*aceE*, Δ*pqo*	l-Valine: 12.4	l-Valine 24.6		0.150	Glucose, acetate	[46]
*C. glutamicum* ATCC13032	*ilvBNC*, *ilvE*	Δ*ppc*, Δ*pyc*, *ICD*^*G407S*^	l-Valine: 8.90			0.220	Glucose	[47]
*C. glutamicum* ATCC13032		Δ*pqo*, Δ*ppc*, Δ*aceE*, Δ*alaT*	l-Valine: 2.64				Glucose	[48]
*C. glutamicum* XQ-9	*ilvBNC*, *leuA*	Δ*ltbR*, Δ*pyc*, Δ*avtA*, inserting terminator before *alaT*	l-Leucine: 28.5				Glucose	[43]
*C. glutamicum* ATCC13032	*leuA* ^R529H/G532D^, *ilvN* ^G20D/I21D/I22F^	Δ*ltbR*, Δ*leuA*, Δ*iolR*, replacing *gltA* promoter with P_*dapA*-L1_	l-Leucine: 6.82	l-Leucine: 23.7	0.564	0.219	Glucose	[33]

The above findings suggested that increasing pyruvate accumulation could efficiently enhance BCAAs production. So far, the main strategies applied to decrease central carbon metabolism consisted of knocking out crucial enzymes or replacing crucial enzyme promoters. One knockout method is λ-red homologous recombination, which can delete a single gene. In the future, CRISPR/Cpf1 might be used to simultaneously knock out several genes (*aceE*, *pyc*, *ppc*, and *pqo*) by designing corresponding sgRNAs. The expression CRISPR/dCas9 or mutated Cpf1 might also be applied to partially or completely block the transcription of crucial enzymes to decrease their intracellular levels.

### Strengthening BCAAs biosynthetic pathways

Enhancing the expression of enzymes involved in BCAAs biosynthetic pathways and weakening the interference of competing pathways are direct strategies that have been applied to centralize metabolic flux for BCAAs production. Table 3 shows the various methods used to strengthen BCAAs biosynthetic pathways and block competing pathways. For example, overexpression of the *ilvBNCD* gene encoding AHAS, AHAIR, and DHAD in *C. glutamicum* ATCC13032 increased l-valine accumulation to 4.92 g/L. The *panBC* and *ilvA* genes were subsequently knocked out to block competing d-pantothenate and l-isoleucine biosynthesis pathways, which further increased l-valine production to 10.7 g/L [49]. Alanine aminotransferase (ATs) can compete with pyruvate to produce l-alanine in *C. glutamicum* [50]. Simultaneous *aceE*, *alaT*, and *ilvA* knockout in *C. glutamicum* ATCC13869 resulted in a 44-fold increase in l-valine production, and overexpressing *ilvBNC*1 further improved l-valine production by 87.6% [45]. Moreover, the *ilvA* and *leuA* promoters were modified to, respectively, decrease TD and IPMS expression; then, strong promoters overexpressed DHAD and TA, which ultimately increased l-valine production to 15.9 g/L [51].

**Table 3:** Enhancement of BCAAs biosynthetic pathways

Host	Overexpression genes	Knockout genes	Titer of BCAAs (shake flask) (g/L)	Titer of BCAAs (fed batch) (g/L)	Productivity (g/(L × h))	Yield (g/g glucose)	Substrate	References
*C. glutamicum* R	*ilvBN*^G156E^, *ilvC*^S34G/L48E/R49F^, *ilvD*, *leuDH*	Δ*ldhA*	l-Valine: 227		7.17	0.410	Glucose	[52]
*C. glutamicum* R JCM 18229	*ilvBN*^G156E^, *ilvC*^S34G/L48E/R49F^, *ilvD*, *leuDH*, *gapA*, *pyk*, *pfk*, *pgi*, *tpi*	Δ*ldhA*, Δ*ppc*, Δ*pta*, Δ*ackA*, Δ*ctfA*, Δ*avtA*	l-Valine: 150			0.573	Glucose	[53]
*C. glutamicum* ATCC13032	*ilvN* ^G20D/I21D/I22F^, replacing *ilvD* promoter with P-*ilvD*M7, replacing *ilvE* promoter with P-*ilvE*M6	Δ*panB*, replacing *ilvA* promoter with P-*ilvA*M1CG	l-Valine: 15.9				Glucose	[51]
*C. glutamicum* ATCC13032	*ilvBNC*, *ilvD*	Δ*ilvA*, Δ*panBC*	l-Valine: 10.7				Glucose	[49]
*C. glutamicum* YILW	*brnFE*	Δ*brnQ*		l-Isoleucine: 29.0			Glucose	[54]
*C. glutamicum* IWJ001	*gnd*, *fbp*, *pgl*		l-Isoleucine: 10.9	l-Isoleucine: 29.0	0.345	0.138	Glucose	[55]
*C. glutamicum* WM001	*phaCAB*		l-Isoleucine: 9.58	l-Isoleucine: 29.8		0.129	Glucose	[56]
*C. glutamicum* JHI3-156	*lrp*, *brnFE*		l-Isoleucine: 3.49	l-Isoleucine: 26.9	0.374	0.122	Glucose	[57]
*C. glutamicum* MH20-22B	*ilvA* ^V323A^, *hom* ^G378E^, introducing the *ilvA*-con promoter mutation, replacing hom promoter with PcspB	Introducing the P*dapA*-C13 promoter mutation	l-Isoleucine: 6.95	l-Isoleucine: 14.3	0.184	0.137	Glucose	[58]
*C. glutamicum* JHI3-156	*zwf*, *ppnK*		l-Isoleucine: 4.10		0.057	0.072	Glucose	[59]
*C. glutamicum* ATCC13869	*lysC*, *hom*, *thrB*, *ilvA*	Δ*ddh*	l-Isoleucine: 3.81				Glucose	[60]
*C. glutamicum* YILW		Δ*alaT*	l-Isoleucine: 2.09	l-Isoleucine: 15.4		0.136	Glucose	[61]
*C. glutamicum* XQ-9	*leuA*, *ilvBN*, *leuDH*, *ilvC*^S34G/L48E/R49F^, *rocG*	Δ*ltbR*, Δ*ilvE*, Δ*gdh*	l-Leucine: 23.3			0.191	Glucose	[62]
*C. glutamicum* FA-1	*aspB*	Δ*ilvE*	l-Leucine: 20.8				Glucose	[63]

Deleting the IPMI and IPMD repressors and the competing pathways for l-isoleucine, l-alanine, d-pantothenate, and lactate production in *C. glutamicum* led to increased l-leucine production (28.5 g/L). The subsequent overexpression of R529H/G532D/L535V IPMS in the knockout strain resulted in 38.1 g/L l-leucine accumulation in a 50-L automated fermenter [64]. Moreover, *C. glutamicum* AN02 with G127D/I197V/R529H/G561D IPMS, IPMI, and IPMD overexpression resulted in more leucine (13.1 g/L) than a strain overexpressing only G127D/I197V/R529H/G561D IPMS [65]. Inserting the terminator with the strength of 40.4 a.u. before *alaT* and replacing native promoters of *ilvBNC* and *leuA* with the strong promoter *P*_*tuf*_ improved l-leucine accumulation to 26.8 g/L [43]. To address the generation of by-products due to the substrate diversity of TA, the *ilvE* gene encoding TA was knocked out, and aspartate aminotransferase (AspB) was overexpressed in *C. glutamicum* FA-1 [63]. That strain produced 82.6% more l-leucine (20.8 g/L) than the original strain, while l-valine production remained unaltered, indicating that AspB has higher specificity for the precursor of l-leucine than l-valine production [63].

Knocking out the *alaT* gene increased the l-isoleucine yield by 17.6% [61], and overexpressing TD, AK, HD, and HK in *C. glutamicum* increased l-isoleucine production 7.6-fold. The l-isoleucine biosynthetic pathway shares some initial steps with the l-lysine and l-methionine pathways, which leads to a shunt of the carbon flux. Based on that, knocking out the *ddh* gene encoding 2,3-diaminopimelate dehydrogenase decreased l-lysine biosynthesis and allocated more carbon resources to l-isoleucine production; this generated an 8% increase in the l-isoleucine yield [60]. Genome promoters that have been mutated to increase TD and HD expression that is free of feedback inhibition and decrease the expression of dihydrodipicolinate synthase (DapA) have resulted in the accumulation of 6.95 g/L l-isoleucine in shaker flasks [58].

### Removing feedback inhibition and transcriptional attenuation of other enzymes

In addition to AHAS, relieving or removing the feedback inhibition and transcriptional attenuation of BCAAs, or intermediate metabolites of other crucial enzymes in *C. glutamicum*, can also alleviate obstacles in BCAAs biosynthetic pathways and further increase the production of corresponding BCAAs. Table 4 shows that introducing the Y553D mutation into IPMS relieved the feedback inhibition of IPMS by l-leucine and increased l-leucine production by 30-fold compared with that in wild-type IPMS [66]. The leading peptide *leuL* (MTSRANLLLLRRGGSQRS*) [30] and the promoter of the *leuA* gene encoding IPMS have been replaced with the P_tuf_ promoter to remove the transcriptional attenuation of l-leucine towards IPMS in *C. glutamicum* and express IPMS (R529H/G532D IPMS) without feedback inhibition [33]. This ultimately resulted in a 50% increase in IPMS activity. Overexpressing R558H/G561D IPMS, which maintained 89% activity towards 2 g/L l-leucine, and replacing the native promoter *leuA* with PCJ7, which is free of transcriptional attenuation, increased l-leucine production 4.2-fold [67].

**Table 4:** Removing the feedback inhibition and transcriptional attenuation of other enzymes

Host	Mutation sites	Titer of BCAAs (shake flask) (g/L)	Titer of BCAAs (fed batch) (g/L)	Productivity (g/(L × h))	Yield (g/g glucose)	Substrate	References
*C. glutamicum* DSM8890	V323A TD	l-Isoleucine: 16.4				Glucose	[68]
*C. glutamicum* IWJ001	P176S/D426E/L575W AHAS, F383V TD	l-Isoleucine: 5.83				Glucose	[69]
*C. glutamicum* JHI3-156	P176S/D426E/L575W AHAS, F383V TD	l-Isoleucine: 5.77	l-Isoleucine: 30.7	0.426	0.120	Glucose	[70]
*C. glutamicum* ATCC13869	A279T AK, G378S HD, F383V TD	l-Isoleucine: 3.53				Glucose	[71]
*C. glutamicum* ATCC14067	V140M/F383A TD	l-Isoleucine: 0.73				Glucose	[72]
*C. glutamicum* ML1-9	R529H/G532D/L535V IPMS		l-Leucine: 38.1	0.794		Glucose	[64]
*C. glutamicum* KCCM11662P	R558H/G561D IPMS	l-Leucine: 13.1				Glucose	[67]
*C. glutamicum* ATCC13032	G20D/I21D/I22F AHAS, R529H/G532D IPMS	l-Leucine: 6.82	l-Leucine: 23.7	0.564	0.219	Glucose	[33]
*C. glutamicum* ATCC13032	Y553D IPMS	l-Leucine: 1.55				Glucose	[66]

The activity of TD is, respectively, reduced by 85% and almost completely lost in the presence of 0.660 and 1.97 g/L l-isoleucine [73]. The V323A TD [68] and H278R/L351S TD [19] mutants that are more resistant to feedback inhibition than the wild type, were created by site-directed mutagenesis to relieve the feedback inhibition of TD by l-isoleucine. The highly conserved site of TD has been targeted, and V140M, F383A, and V140M/F383A TD mutants were created. The specific activity of V140M and V140M/F383A TD was 1.5-fold higher than that of wild-type TD. Thus, F383A TD increased the l-isoleucine titer from 0.470 to 0.730 g/L [72].

l-threonine is a crucial intermediate in the pathway of l-isoleucine production, as it induces feedback inhibition of the enzymes AK, HD, and HK that are involved in l-threonine production. l-threonine inhibits HD and HK activity by binding to allosteric sites on AK and HD and inhibits the activity of HK by competing with native substrates for its active sites [74]. The mutants A279T AK and G379S HD created by site-directed mutagenesis completely and partially resisted the feedback inhibition of l-threonine, respectively [71]. The A20G mutation introduced into HK induces activity comparable with that in wild-type HK and substantial ability to resist feedback inhibition by l-threonine. Meanwhile, this mutant has a 5.3-fold lower Ki than the wild type. Structural analysis of HK revealed that changes in the van der Waals forces in A20G HK affected the affinity of l-threonine for active sites [74]. Based on this, AK, HD, and HK expressed without feedback inhibition in *C. glutamicum* have contributed to increased yields of l-lysine, l-threonine, and l-isoleucine [60]. Site-directed mutagenesis has achieved the efficient release of feedback inhibition that further enhanced BCAAs yields.

### Improving the supply of cofactors for crucial enzymes

Balancing the intracellular reducing power derived from NADH and NADPH is significant for maintaining cell homeostasis and biosynthesizing target compounds. The cofactor NADPH is essential for the enzymes, ASD, HD, AHAIR, and TA, which are involved in BCAAs biosynthetic pathways [75, 76]. The absence of nicotinamide nucleotide transhydrogenase complicates the reversible conversion of NADH to NADPH in *C. glutamicum* [77, 78], leading to a limited supply of NADPH and further affecting BCAAs production. Therefore, increasing the supply of NADPH to reinforce crucial enzyme reactions is a key step in improving BCAAs production. Two pathways can be used to produce NADPH. One is via the NAD (H) kinase catalysis of NAD^+^ and/or NADH to produce NADP^+^ and/or NADPH [79, 80], and the other is via the dehydrogenase catalysis of NADP^+^ to produce NADPH [81]. The glucose-6-phosphate (G6PD) and 6-phosphogluconate (6PGDH) dehydrogenases in *C. glutamicum* are mostly derived from the pentose phosphate pathways [82]. Therefore, the NAD kinase Ppnk has been overexpressed in a strain that increased l-isoleucine production to 32.4 g/L [69, 70]. Co-expressing Ppnk with G6PD improved the l-isoleucine yield by 85.9% [59]. Furthermore, enhancing the pentose phosphate pathway by overexpressing 6PGDH, 6-phosphogluconolactonase (PGL), and 1,6-bisphosphatase (FBPase) increased the l-isoleucine titer from 6.60 to 10.9 g/L in shaker flasks [55].

Switching NADPH-dependent to NADH-dependent reactions is an alternative approach because the supply of NADH in *C. glutamicum* is sufficient for NADH-dependent reactions to proceed. To convert AHAIR and TA into NADH-dependent enzymes in *C. glutamicum*, AHAIR was modified by introducing the S34G/L48E/R49F mutations and replacing TA with leucine dehydrogenase (LeuDH) from *Lysinibacillus sphaericus*. The engineered strain produced 227 g/L l-valine by overexpressing AHAS free of feedback inhibition [52]. Subsequently, NADH-dependent AHAIR was integrated with AHAS free of feedback inhibition into the genome of a *C. glutamicum* strain with an enhanced glycolysis pathway and deleted pathways of biosynthetic by-products to achieve an l-valine yield of 150 g/L [53]. In addition, the overexpression of NADH-dependent AHAIR, LeuDH from *Lysinibacillus sphaericus,* and glutamate dehydrogenase (GDH) from *Bacillus subtilis* in *C. glutamicum* XQ-9Δ*ltbR*/*leuAilvBNC* increased l-leucine production to 23.3 g/L [62].

Overexpression of crucial enzymes and deletion of competing pathways have been focused upon to strengthen BCAAs biosynthetic pathways. Replacing native promoters to increase or decrease target enzyme expression can also enhance the metabolic flux of BCAAs biosynthetic pathways. The strategies described above were based on published studies. Elucidation of the details of BCAAs biosynthetic pathways still requires a deep and comprehensive analysis. A dynamic metabolic network should be constructed, and a real-time monitoring system should be adopted to identify other rate-limiting or competing steps. Thereafter, novel strategies could be designed and constructed to further enhance BCAAs production. In addition, the substrate diversity of AHAIR, DHAD, and TA should be determined via mutagenesis or by replacing them with their isozymes with substrate specificity to realize the production of individual BCAAs. For example, transaminase TyrB from *E. coli*, which catalyzes only 2-ketoisocaproate to produce l-leucine, could replace the substrate-diverse transaminase TA in *C. glutamicum* to allocate more carbon source for the sole production of l-leucine [63].

## Regulation of BCAAs transporters

The two-component transporter protein in *C. glutamicum*, BrnFE, transports BCAAs and l-methionine into the extracellular milieu, whereas BrnQ transports BCAAs into cells [83]. Complexes comprising BCAAs and the global regulatory factor Lrp bind to the interval sequence between *lrp* and *brnFE* to activate BrnFE expression [83, 84]. Therefore, regulating the expression of Lrp can regulate the BCAAs transport and further influence BCAAs production. For example, Lrp overexpressed in *C. glutamicum* ATCC13869 increased l-valine and l-isoleucine production by 16- and 8.9-fold, respectively [45]. Furthermore, overexpressed Lrp, BrnFE, AHAS, and AHAIR in *C. glutamicum* ATCC13869Δ*aceE*Δ*alaT*Δ*ilvA* has yielded 28.5 g/L l-valine in shake flask [45]. Deleting BrnQ and overexpressing BrnFE increased the titer from 20.2 to 29.0 g/L in a strain producing l-isoleucine [54], whereas co-expressed Lrp and BrnFE in *C. glutamicum* JHI3-156 increased l-isoleucine production by 63% in shaker flasks and by 72% in fed-batch fermentation [57] (Table 3).

**Fig. 2:**
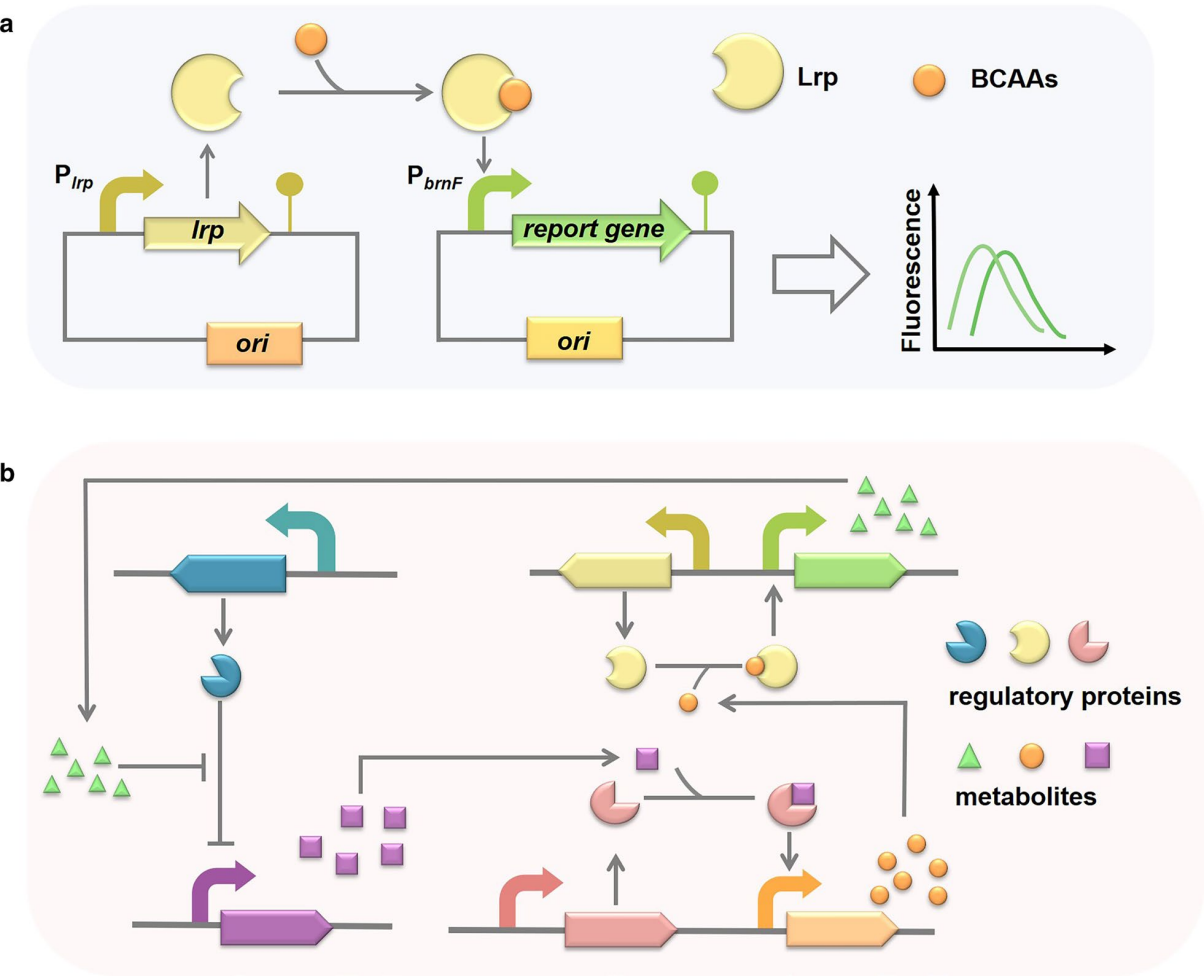
**a** Schematic illustration of *lrp*-P_*brnFE*_ biosensor responding to BCAAs. With the accumulation of BCAAs in the cell, Lrp responds to BCAAs and binds to P_*brnF*_ region and activates the expression of report gene. **b** The metabolites activate biosensor system to construct the dynamically regulation network, so as to achieve the high-level biosynthesis of value-added compounds

Based on the above regulation mechanisms, the *lrp-brnEF* regulation system has been engineered to establish a BCAAs biosensor that could convert specific concentrations of BCAAs into easily detectable YFP signals [85]. The BCAAs concentration in the biosensor was linear with fluorescence signal intensity, and strains producing abundant l-valine were screened by fluorescence activated cell sorting (FACs) [86]. The *lrp-brnEF* regulation system has been applied to dynamically regulate the metabolic pathway involved in producing the synthetic unnatural amino acid 4-hydroxyisoleucine (4-HIL) [87]. Subsequently, the promoter P_brnFE_ in the *lrp-brnEF* regulation system was modified to the stronger promoter P_brnFE_N, which was used to regulate the transcription of l-isoleucine dioxygenase (IDO), that could convert l-isoleucine into 4-HIL in the presence of α-KG and oxygen. Meanwhile, the supply of α-KG and oxygen was coordinately modulated via this regulation system, which finally produced 19.9 g/L 4-HIL [88]. In general, regulating BCAAs transporter expression efficiently enhances BCAAs production. The *lrp-brnEF* regulation system can be used to establish biosensor to screen productive strains (Fig. 2a). First, BCAAs produced in the culture could bind to regulatory protein Lrp which under the regulation of P_*lrp*_. Then, the generated complex could activate the promoter P_*brnF*_, which regulates the transcription of report gene such as *gfp*, *yfp* and *rfp*. Detection of report protein could directly reflect the concentration of intracellular BCAAs. This biosensor system might be applied for screening BCAAs high-productive strains. In addition, *brnFE-lrp* system could also be used for establishing dynamic metabolic network to increase the biosynthesis of value-added compounds. As shown in Fig. 2b, different metabolites bind to corresponding regulatory proteins to activate or inhibit the expression of downstream genes. The cascade system regulated by the concentrations of metabolites could realize the dynamic regulation of target compounds production. Meanwhile, the construction of dynamic regulation network could realize the optimal distribution of BCAAs in cell growth and products biosynthesis.

## Bioinformatics analysis of strains producing BCAAs

Analysis of strains producing BCAAs using genomics, transcriptomics, proteomics, and metabolomics could reveal significant information for further host modification to maximize BCAAs production. A comparison of intracellular metabolites and BCAAs production by wild-type *C. glutamicum*, *C. glutamicum* XV (producing l-valine), and *C. glutamicum* CP (producing l-leucine) [89] revealed a more active pentose phosphate pathway in the XV or CP strains than the wild type, indicating that these strains could produce more NADPH than the wild type. *C. glutamicum* XV and CP were obtained via multiple rounds of random mutagenesis [65], and these two strains are accessible at the China General Microbiological Culture Collection Center with the identifiers of CGMCC11425 (CP) and CGMCC 1.15672 (XV), respectively [89]. The transcription of most genes in l-valine and l-leucine biosynthetic pathways is up-regulated in the XV and CP strains [89]. A subsequent comparison of the three strains by time-series metabolomics identified metabolic differences mainly found in central carbon metabolism and stress resistance. *Corynebacterium glutamicum* XV has evolved more pyruvate for l-valine synthesis and accumulated trehalose to resist environmental stress, whereas *C. glutamicum* CP has high membrane permeability that could improve the efficiency of extracellular l-leucine transport [90]. A comparison of transcriptomic and proteomic data from *C. glutamicum* VWB-1, an industrial l-valine producer, and wild type *C. glutamicum* ATCC13869 uncovered significantly different transcriptional levels of 1,155 genes and the abundance of 96 proteins between them [91]. Specifically, transcription of the *ilvBN*, *ilvC*, *ilvD*, and *ilvE* genes crucial to the l-valine biosynthetic pathway was upregulated, whereas that of the *leuB* and *ilvA* genes crucial to the l-leucine and l-isoleucine biosynthetic pathways was downregulated. Thus, the metabolic flux of l-valine pathway was enhanced, and the competing pathway was blocked to increase the yield of l-valine. Analysis of the *C. glutamicum* transcriptome uncovered the transcriptional attenuators, *trpL*, *ilvL*, and *leuL*, which are, respectively, involved in l-tryptophan, l-valine, and l-leucine production. This was the first use of transcriptome datasets to accurately describe attenuation locations and attenuator structures, and it laid a foundation to relieve the effects of attenuation and further improve BCAAs yield [30]. In general, *C. glutamicum* bioinformatics analysis has offered some explanations for elevated BCAAs production and some evidence to support the engineering strategies described above. In addition, bioinformatic data could be applied to establish other regulation strategies that might enhance the production of BCAAs or their derivatives. Combining bioinformatics technology with synthetic biology has significant value for strain modification, which could provide a basis and guide the maximal yields of target compounds.

## Conclusions and perspectives

**Fig. 3:**
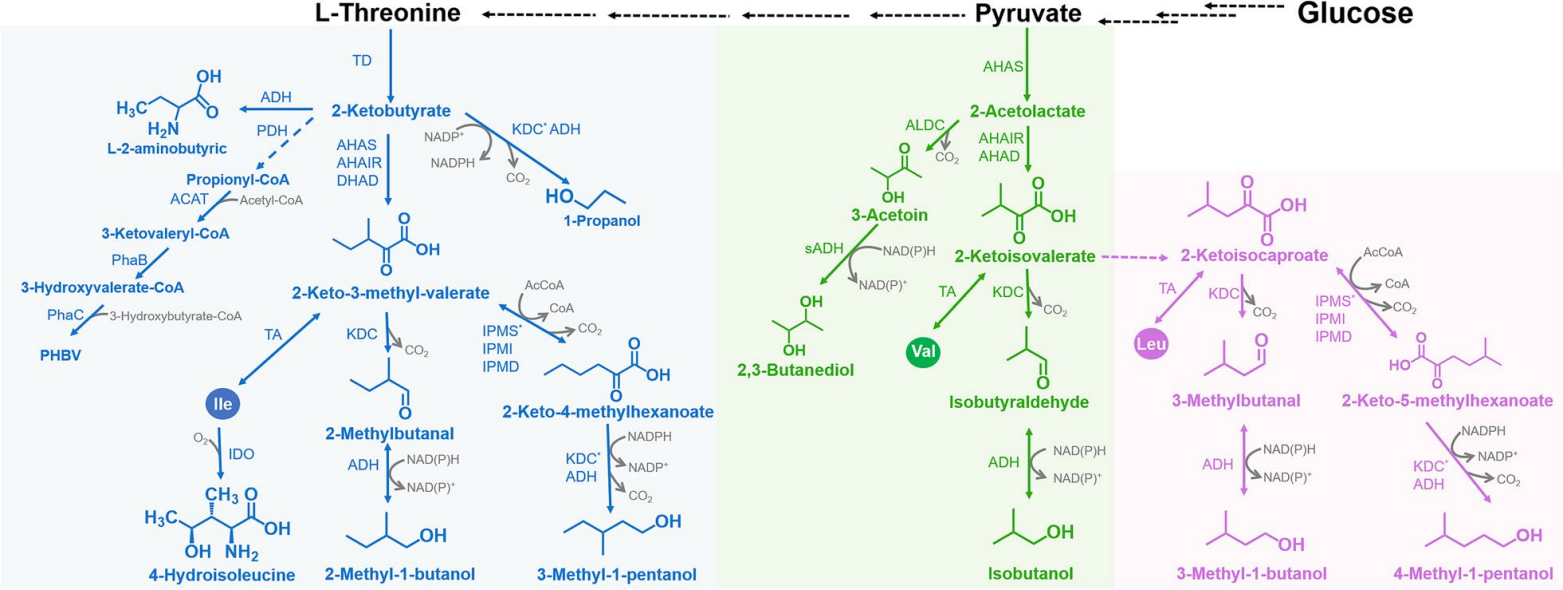
Biosynthetic pathways of BCAAs derivatives in *C*. *glutamicum*. The blue areas represent derivatives related to the l-isoleucine metabolic pathway, the green areas represent derivatives related to the l-valine metabolic pathway, and the pink areas represent derivatives related to the l-leucine metabolic pathway. ADH: alcohol dehydrogenase; PDH: pyruvate dehydrogenase; ACAT: Acetyl-CoA acetyltransferase; PhaB: acetoacetyl-CoA reductase; PhaC: poly(3-hydroxybutyrate) polymerase; PHBV: Poly(3-hydroxybutyrate-co-3-hydroxyvalerate); KDC: keto acid decarboxylase; IDO: isoleucine dioxygenase; ALDC: acetolactatedecarboxylase; sADH, secondary alcohol dehydrogenase

Removing feedback inhibition and/or the transcriptional attenuation of some crucial enzymes is significant for enhancing BCAAs production. However, the lack of clarity about the underlying mechanisms prevented the complete elimination of feedback inhibition and transcriptional attenuation. Therefore, the crystal structures, molecular docking complexes, and mechanisms of feedback inhibition and/or the transcriptional attenuation of crucial enzymes were analyzed. With better understanding of the involved mechanisms, mutations that could induce a target enzyme to completely delete feedback inhibition and/or transcriptional attenuation could be rationally designed and introduced into target enzymes to address the problems of intermediates or low yields caused by BCAAs accumulation. The structural similarity of BCAAs precursors and the promiscuity of enzymes involved in BCAAs biosynthetic pathways resulted in the mixed production of three BCAAs, which complicated BCAAs separation and purification. The enzymes shared in three BCAAs biosynthetic pathways needed to be crystallized, and the catalytic mechanisms of different precursors required exploration. Thus, substrate-specific enzymes were created through directed mutagenesis and applied to the production of individual BCAAs. Construction of a computer algorithm-assisted metabolic network model might be an efficient and economical method of determining the rate-limiting points of BCAAs metabolic pathways. Thereafter, a series of modifications could be created to address the rate-limiting points and generate strains suitable for industrial-scale BCAAs production. Such strains might be used to biosynthesize BCAAs derivatives such as isobutyraldehyde [92], isobutanol [93], and 3-methyl-1-butanol [94], which share precursors with BCAAs (Fig. 3). Meanwhile, developing strains producing BCAAs might provide guidance for constructing other strains that could produce valuable compounds.

## Data Availability

Data sharing is not applicable to this article as no datasets were generated or analyzed during the current study.
